# Visuospatial Function in Women with Premenstrual Dysphoric Disorder

**DOI:** 10.3390/jcm13072004

**Published:** 2024-03-29

**Authors:** Ajna Hamidovic, Soojeong Cho, Shahd Smadi, John Davis

**Affiliations:** 1 College of Pharmacy, University of Illinois at Chicago, Chicago, IL 60612, USA; scho204@uic.edu; 2 College of Medicine, University of Illinois at Chicago, Chicago, IL 60612, USA; ssmadi2@uic.edu (S.S.); davisjm@uic.edu (J.D.)

**Keywords:** visuospatial, mental rotation, estradiol, premenstrual dysphoric disorder

## Abstract

**Background/Objectives**: Premenstrual dysphoric disorder (PMDD) is an understudied psychiatric condition affecting reproductive-age women who experience negative mood in the luteal phase of the menstrual cycle. Cognitive functions in PMDD are not well understood as patients have been tested in the luteal phase. This may confound study results due to noted emotional interferences, as well as the potential opposing effects of the sex hormones estradiol and progesterone. In the present study, we evaluated visuospatial function in the follicular phase in women with PMDD and healthy controls, and further examined the effect of estradiol as research into the hormonal mediation of visuospatial function in reproductive-age women has produced mixed results. **Methods**: To this end, we analyzed estradiol concentrations using the gold standard mass spectrometry. Serum samples were collected in the early follicular and mid/late follicular subphases when estradiol is low and high, respectively, while progesterone is low and steady. We assessed visuospatial function using the classic mental rotation task. **Results**: Women with PMDD had a higher mental rotation total score (t = 2.17; *p* < 0.05). The addition of six demographic, biological, and anthropomorphic variables in a hierarchical fashion accounted for 45.3% of the total variance in the final model with diagnosis remaining statistically significant (t = 4.36; *p* < 0.001). Estradiol did not mediate the group difference and was not significantly associated with visuospatial function. **Conclusions**: The present results provide support for new research directions into the potential biological mechanisms that underlie the pathophysiology of PMDD, represented as enhanced visuospatial ability in women with PMDD in the follicular phase. We review the theory that PMDD is a disorder of the enhanced excitation-to-inhibition ratio, with a focus on findings to date from brain imaging research.

## 1. Introduction

It is estimated that approximately 5% of reproductive women have premenstrual dysphoric disorder (PMDD) [1], a debilitating psychiatric condition characterized by the expression of negative mood symptomatology in the luteal phase of the menstrual cycle. Along with affective and physical symptoms, women with PMDD may experience luteal phase impairments in cognition that impact their occupational and other daily functions [2,3].

Hormones from the steroid hormone biosynthesis pathway, such as the sex hormones estradiol and progesterone, significantly affect cognitive functions [4]. Sex hormones vary across the menstrual cycle; an infradian rhythm that is broadly divided into follicular and luteal phases. The follicular phase, which starts on the day of menstrual bleeding and ends with ovulation, is followed by the luteal phase, which ends prior to the start of the subsequent menses. In the early follicular *sub*phase, estradiol and progesterone are low. Estradiol then rises in the mid-follicular subphase to its peak in the late follicular, or periovulatory, subphase. Both estradiol and progesterone trajectories follow inverted U-shaped trajectories through the early, mid-, and late luteal subphases.

Hormonal modulations can have profound effects on cognition, given the wide distribution of estrogen (ER-α and ER-β) and progesterone (PR-A and PR-B) [5,6] receptors in cognitively relevant brain regions. For example, ovariectomized rats have reduced dendritic spine density in neurons of the hippocampus and the prefrontal cortex [7]. Following the add-back of estradiol, there are spinal density increases in these areas along with memory improvements [8]. It is, however, not well understood whether the effects of profound shifts in hormone levels, such as ovariectomy and exogenous hormone administration, on memory and cognition translate to the more subtle hormonal changes across the menstrual cycle. 

It is critical to examine this question in the context of the fine-grained, subphase menstrual cycle context, and not phase (follicular vs. luteal) contrasts as there are confounding effects in the latter comparison. The luteal phase is marked by elevations in both estradiol and progesterone, and since progesterone can both enhance as well as antagonize the estradiol enhancement of spinal density [9], this may mask any potential effects of estradiol. 

The phase contrast framework may have contributed to the conclusion that there is insufficient evidence to support menstrual cycle phase differences in visuospatial function [10]. The premise of examining this particular aspect of cognition was based on the finding that men perform better on visuospatial function tasks [11]. As such, it was hypothesized that the sex hormones estradiol and/or progesterone may decrease visuospatial ability; hence, an improvement would be observed at the beginning of the follicular phase of the menstrual cycle when sex hormone levels are low. Nine out of twelve studies, however, compared the follicular vs. luteal phases; however, this approach may be confounded as described above. The three studies that avoided the potential effects of progesterone on estradiol activity in the luteal phase and assessed only the follicular phase did not find timepoint differences when testing the participants during menses and another time on the 11/12th day of the menstrual cycle [12], between days 3 and 7 of the menstrual cycle [13], and between days 1 and 3 and again between days 12 and 20 [14].

These studies drew speculative inferences about the effects of estradiol based on the cycle day, which may be an erroneous approach as phase duration and estradiol levels are significantly variable both within and between women [15,16]. Hence, assessing estradiol concentration using mass spectrometry (MS), which is the gold standard [17] for measuring the concentration of hormones from the steroid biosynthesis pathway, the first objective of the present study was to examine whether estradiol levels correlate with visuospatial function in reproductive-age women. We completed this analysis by testing study participants in the early follicular and mid-late follicular subphases of the menstrual cycle, and adjusted the analysis using serum progesterone, also measured using MS.

The second objective of the present study was to assess whether visuospatial function differs in women with PMDD relative to healthy controls in the follicular phase of the menstrual cycle and whether this potential difference is mediated by serum estradiol concentrations. Although cognitive impairment occurs in the luteal phase, we focused on the follicular phase given the need to assess cognition in PMDD when women are not affected by emotional lability observed in the luteal phase. 

## 2. Materials and Methods

### 2.1. Study Procedures 

The present study is part of a project examining the trajectories of neuroactive steroid hormones in women with PMDD and healthy controls, and the effects of these hormones on heart rate variability, cognition, sleep, and food cravings. Details of the project are described in detail in Hamidovic et al. (2022) [18]. In summary, reproductive-age women with regular menstrual cycles were enrolled if they were not illicit drug users (urine test-verified), smokers, heavy alcohol drinkers, or prescription medication users (including hormonal forms of birth control). Women with a lifetime psychiatric disorder, except anxiety and depression (based on the Structured Clinical Interview for DSM Disorders (SCID)), and current (i.e., within the past 12 months) DSM-5 Major Depressive Disorder or an anxiety disorder (based on SCID) were excluded from the study. Once enrolled, the participants filled out the Daily Record of Severity of Problems (DRSP) survey [19] over 2–3 menstrual cycles, which was used for assigning PMDD diagnosis in the present study [20]. Women were assigned to the PMDD group if they had a 30% or greater increase in 5 or more symptoms (one had to be affective in nature) on days −7 to −1 relative to days +6 to +12 of the menstrual cycle [21]. 

In the last menstrual cycle of the study, the participants attended 8 menstrual cycle visits during which, among other measures, we collected serum samples for the analysis of neuroactive steroid hormone levels according to the Biocycle method [16], as described by Hamidovic et al. (2022) [18]. For example, a woman with a 28-day cycle (based on the previous cycle) was scheduled to attend visits on days 2 (early follicular), 7 (mid-follicular), 12 (periovulatory 1), 13 (periovulatory 2), 14 (periovulatory 3), 18 (early luteal), 21 (mid-luteal), and 27 (late luteal), corresponding to visits 1–8, respectively. Study participants in the present study were randomized to complete the mental rotation task on visit 2 or one of the 3 periovulatory visits to permit adequate follicular phase estradiol variability.

### 2.2. Study Measures

Serum Progesterone and Estradiol. Serum samples were analyzed at the University of Illinois, Chicago Mass Spectrometry Core, using the AB SCIEX 6500 QTRAP mass spectrometer (Sciex, Framingham, MA, USA) coupled with the Agilent 1290 UPLC system. Details of mass spectrometry experiments are described by Hamidovic et al. (2023) [22]. The accuracy of the calibration standard was within the acceptable range of 15%. For progesterone, the limit of detection (LOD) was between 0.5 and 1.5 pg, while for estradiol, the LOD was between 0.05 and 0.25 pg.

Mental Rotation. As in previous studies assessing the effect of estradiol on visuospatial function [23], we used the classic mental rotation task by Vandenberg and Kuse (1978) [24]. Most researchers have used this or similarly related difficult versions to meet the requirement that a visuospatial task should be sufficiently difficult, with three-dimensional instead of two-dimensional depictions, and large, as opposed to small, angular disparities [23,25]. In the task, a target three-dimensional cube is presented along with four alternative figures at different angles. The goal is to select which two of the four alternatives are the same as the target figure. The MRT was scored using the 1-point system [26] in which an item was allotted 1 point if both rotated figures were correctly identified. Each participant was given 8 min to complete 24 problems on paper.

### 2.3. Data Analysis

We first compared the demographic and anthropomorphic characteristics of the study groups (PMDD vs. healthy control) using the Chi-Square (or Fisher’s Exact) test for categorical variables and the analysis of variance (ANOVA) for continuous variables (Table 1). While the distribution of the mental rotation total score was normal, one participant had 0 as the final score; hence, this participant’s data were removed from the analysis. Next, we completed linear regression analysis to evaluate the significance of the association between the mental rotation total score and estradiol. In this analysis, we ran an additional model, adjusting for progesterone concentration. Our second evaluation was the hierarchical linear regression analysis evaluating the significance of the association between the mental rotation total score (outcome) and diagnosis (predictor) (Model 1). We next entered age and age of menarche in step 2, followed by BMI (coded as underweight/normal weight (BMI ≤ 24.9) or overweight (BMI > 25)) and estradiol in Model 3. In Model 4, we entered race as a factor variable. In the final analysis, we ran a linear regression with the diagnosis–estradiol interaction (with the main effects of each) as a predictor of the mental rotation total score. We formally tested the distribution of residuals for all models using the “shapiro.test” function in R. We set the statistical significance of all analyses at *p* ≤ 0.05. 

## 3. Results

The present analysis included 17 healthy participants and 12 participants with PMDD. They were 25.89 years old on average, and ~60% were students. About half of the participants were white. The mean age of menarche was 12.17, and the average BMI was 25.03. As indicated in Table 1, there were no statistically significant demographic or anthropomorphic differences between the two groups. 

**Figure 1:**
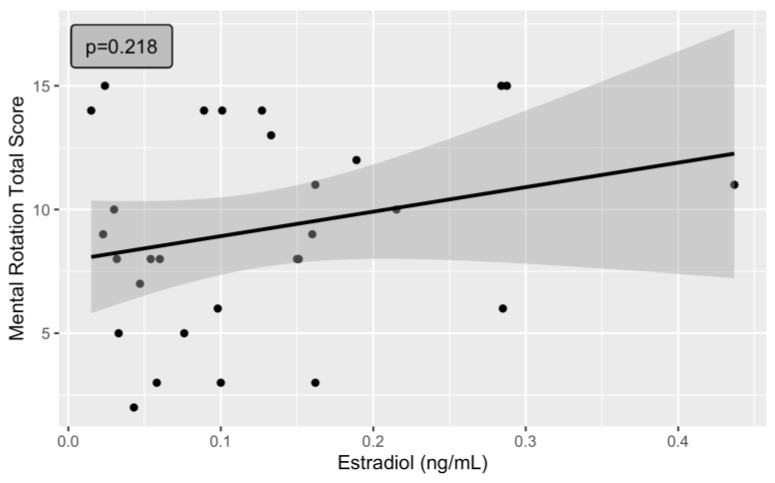
Absence of a significant association between mental rotation total score and estradiol levels in the follicular phase of the menstrual cycle.

The linear regression model examining the association between estradiol and total score on the mental rotation task did not indicate significance (*t*-value = 1.263; *p*-value = 0.218) (Figure 1). The Shapiro test assessing the distribution of residuals was not statistically significant (W = 0.953, *p*-value = 0.242). Adjusting the model for progesterone did not result in statistical significance for estradiol (*t*-value = 1.407; *p*-value = 0.172). Likewise, progesterone was not significantly associated with the total mental rotation score (*t*-value = −0.923; *p*-value = 0.365).

**Figure 2:**
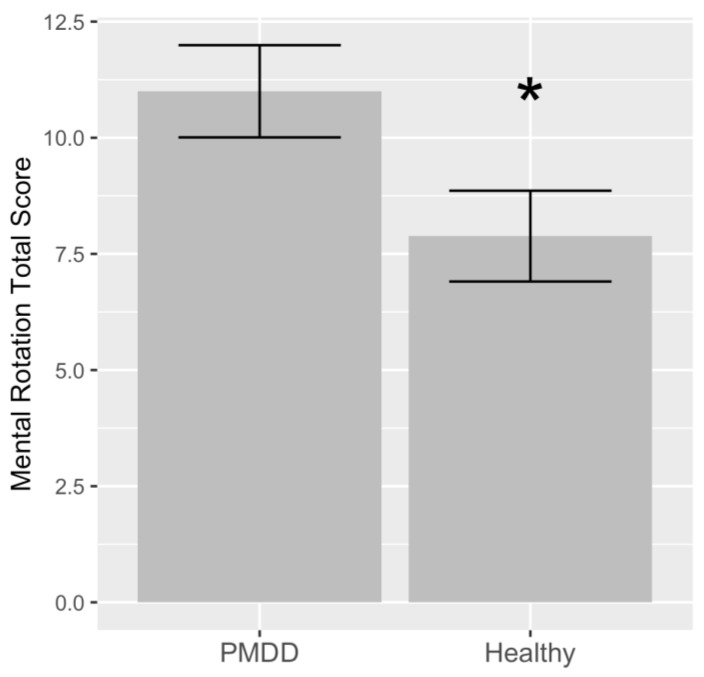
Significantly lower mental rotation total score in healthy study participants relative to the study participants with PMDD (*p* < 0.05). This significance remained after the adjustment for additional covariates (*p* < 0.001) (Table 2). * *p* < 0.05. Variance is displayed as the standard error of the mean.

Detailed results of the hierarchical analysis are presented in Table 2. Model 1 of the hierarchical linear regression showed that women with PMDD had a higher mental rotation total score (*t* = 2.17; *p* < 0.05) (Figure 2), with diagnosis accounting for 11.7% of the variation. Adjusting for age and age of menarche in Model 2 showed that diagnosis remained significant (*t* = 2.42; *p* < 0.05), while age (*t* = 1.12; *p* = 0.272) and age of menarche (*t* = 1.34; *p* = 0.192) were not significantly associated with the mental rotation total score. Model 2 accounted for 14.9% of the total variance. Adjusting for age, age of menarche, BMI, and estradiol levels in Model 3 indicated that the diagnosis is significantly associated with the mental rotation total score (*t* = 3.44; *p* < 0.01). In this model, age was associated (*t* = 2.13; *p* < 0.05), while age of menarche (*t* = 0.73; *p* = 0.470), BMI (*t* = −1.52; *p* = 0.140), and estradiol (*t* = 1.58; *p* = 0.127) were not associated with the mental rotation total score. Model 3 accounted for 37.9% of the total variance. In the final model, diagnosis was significant (*t* = 4.36; *p* < 0.001). In this model, age (*t* = 0.88; *p* = 0.388), age of menarche (*t* = 1.34; *p* = 0.196), BMI (*t* = −1.44; *p* = 0.168), estradiol (*t* = 1.86; *p* = 0.080) and race (with white as a reference) were not associated with the mental rotation total score. The final model accounted for 45.3% of the total variance. The results of the Shapiro–Wilk normality tests on individual model residuals were not significant for any of the four models assessing the association between diagnosis and the mental rotation total score. 

The final analysis showed that the mental rotation total score did not differ by group according to the estradiol concentration, as the diagnosis–estradiol association was not significant (*t* = 1.29; *p* = 0.209). Estradiol (*t* = −0.16; *p* = 0.869) was not statistically significant, while diagnosis remained significant (*t* = 2.36; *p* < 0.05).

## 4. Discussion 

The results of the present study indicate that estradiol is not significantly associated with visuospatial performance in reproductive-age women in the follicular phase of the menstrual cycle when estradiol significantly varies while progesterone stays low and steady. Furthermore, the results show that women with PMDD have better mental rotation performance relative to healthy controls. This finding held after the adjustment for a number of important demographic and anthropomorphic covariates. Finally, the group difference in visuospatial performance identified here was not modulated by circulating estradiol levels. 

The rationale for examining estradiol’s effects on visuospatial function stems from the finding that men outperform women in visuospatial performance by approximately 0.6 standard deviations [27]. Hence, several studies of varying designs—including both direct measurements of estradiol as well as menstrual cycle phase evaluations as proxies of underlying estradiol concentrations—have been completed. To this effect, a meta-analysis [10] contrasting the follicular vs. luteal phase in visuospatial performance found no effect of the phase (standardized mean difference = 1.61 (95% CI −0.35 to 3.57)), though a number of limitations were present in the analyzed studies, including the possible confounding effect of luteal phase progesterone on the potential effects of estradiol. Moreover, studies to date [13,25,28,29,30] analyzed estradiol using immunoassay techniques, which are considered unreliable due to poor specificity [31] to distinguish similar compounds from the steroid biosynthesis pathway. Assays based on mass spectrometry are the gold standard method for the quantification of estradiol [32] and are implemented in Clinical Laboratory Improved Amendments (CLIA)-certified laboratories. Using mass spectrometry as a validated method for estradiol measurement in a menstrual cycle phase devoid of high and variable progesterone, the results of the present analysis indicate no significant effect of estradiol on mental rotation performance. The present results are in line with the meta-analysis [10], showing no effect of menstrual cycle phase, which was implemented to serve as a proxy of sex hormones. 

This result was consistent across the study groups, as the interaction between serum estradiol and diagnosis on the mental rotation total score was not statistically significant, while a significant main effect of the diagnosis was identified. The finding that women with PMDD have better visuospatial function may reflect a distinct tone in the areas processing mental rotation, including the occipital lobe [33,34,35]. Interestingly, levels of gamma-aminobutyric acid (GABA)—the main inhibitory neurotransmitter in the human central nervous system—are reduced in the occipital lobe in the follicular phase of the menstrual cycle in women with PMDD relative to healthy controls [36], supporting the notion that the underlying pathophysiology of PMDD is related to the increased ratio of neuronal excitation-to-inhibition. With respect to the occipital lobe tone, the increased excitation in PMDD was observed in a phase-specific manner, as occipital GABA levels increased in women with PMDD from the follicular to the luteal phase, while the opposite pattern was identified in healthy controls [36]. Furthermore, by recording magnetoencephalographic (MEG) gamma oscillations (~30–90 Hz), Manyukhina et al. (2023) [37] found that neuronal excitability in the visual cortex is consistently elevated across the menstrual cycle in women with PMDD in the form of visual gamma response (GR) power. The primary visual cortex concurrently mediates both the perception of an external input and the internal generation of a transformed representation, which comprises mental rotation [38]. Hence, the results of the present study may reflect enhanced tone in this brain region in PMDD, though meta-analytic findings have identified additional consistent networks of foci that are active during mental rotation [35]. Hence, excitatory tones in other regions, as well as morphological differences, may be further examined. As an example, the parietal lobe tissue proportion in women is greater than in men [39], which links to the noted sex differences in mental rotation test performance [40]. Apparently, these differences extend to the prenatal period [41]. Hence, developmental origins of differences between women with PMDD and healthy controls may be present [42], just as there are significant sex differences that are attributed in part to brain morphology.

The conduct of the present study was severely impacted by the COVID-19 pandemic, resulting in a small sample size. Hence, the results presented here should be cautiously interpreted in the absence of confirmation from a larger study. Nonetheless, a number of potentially confounding factors were tightly controlled as the study participants did not have present co-occurring mental illnesses, take prescription medications (including hormonal forms of birth control), smoke, or take illicit drugs. 

Luteal phase PMDD-related changes in affect or fatigue may affect efforts to complete the mental rotation task. Hence, we conducted the present study in the follicular phase, though a better design would have involved including an additional mental rotation evaluation in the luteal phase in a between-subject fashion to avoid carryover effects. Due to budgetary constraints, we were not able to complete this aspect of the study. Moreover, as previous exposure to the test in within-subject designs may result in carryover effects [23], the importance of strategy and experience cannot be overlooked, presenting caution in drawing definitive conclusions before replication studies are performed. 

## 5. Conclusions

In this study, we reported on the visuospatial ability of women with PMDD and healthy controls and its relationship with estradiol in the follicular phase. This result potentially opens new directions for research into biological factors that underlie the pathophysiology of PMDD; represented as an enhanced visuospatial ability in women with PMDD. Although estradiol does not play a role, other hormones, as well as other factors, such as brain anatomy and environmental and genetic factors, may be investigated with an overarching goal of gaining a better insight into the pathophysiology of PMDD, thereby facilitating drug discovery. 

## Figures and Tables

**Table 1:** Demographic characteristics according to study groups.

Demographic Variable	Category	Diagnostic Category		
		Healthy (*n* = 17)	PMDD (*n* = 12)	*p*-Value
Age		26.23 (5.27)	24.75 (4.95)	0.45
Race	White	5	5	0.73
	Black or African American	4	1	
	American Indian or Alaska Native	0	1	
	Asian	5	3	
	Native Hawaiian or Other Pacific Islander	0	0	
	More than one race	1	0	
	Unknown/do not want to specify	2	2	
Ethnicity	Hispanic	4	5	0.42
	Non-Hispanic	11	7	
	Unknown/do not want to specify	2	0	
Student status	Yes	7	5	0.14
	No	5	12	
Age of menarche		12.25 (1.57)	12.08 (1.164)	0.76
BMI		24.17 (4.81)	26.18 (4.76)	0.28

**Table 2:** Variables for hierarchical linear regression analysis evaluating the significance of the association with mental rotation total score (outcome).

Model	Variable	Adjusted R^2^	Estimate	SE	*t* Value	Pr(>|t|)	Significance
1	Diagnosis (PMDD)	0.1177	3.118	1.433	2.17	0.0385	*
2	Diagnosis (PMDD)	0.1492	−3.455	1.424	2.42	0.0228	*
	Current age		0.156	0.139	1.12	0.2723	
	Age of menarche		0.693	0.517	1.34	0.1923	
3	Diagnosis (PMDD)	0.3308	4.552	1.321	3.44	0.0023	**
	Current age		0.283	0.133	2.13	0.0441	*
	Age of menarche		0.367	0.500	0.73	0.4705	
	BMI		−2.196	1.437	−1.52	0.1407	
	Estradiol		10.10	6.372	1.58	0.1271	
4	Diagnosis (PMDD)	0.4537	5.566	1.275	4.36	0.0004	***
	Current age		0.127	0.144	0.88	0.3889	
	Age of menarche		0.726	0.540	1.34	0.1965	
	BMI		−2.019	1.402	−1.44	0.1681	
	Estradiol		13.24	7.121	1.86	0.0802	.
	Race (African American)		2.594	1.792	1.44	0.1660	
	Race (American Indian/Alaska Native)		−5.829	3.540	−1.64	0.1179	
	Race (Asian)		−1.135	1.849	−0.61	0.5474	
	Race (more than one race)		4.603	3.550	1.29	0.2120	
	Race (unknown/do not want to specify)		−2.600	1.977	−1.31	0.2060	

Significance codes: *** 0.001; ** 0.01; * 0.05; . 0.1.

## Data Availability

The data presented in this study are available on request from the corresponding author.

## References

[1] Gehlert S., Song I.H., Chang C.-H., Hartlage S.A. (2008). The prevalence of premenstrual dysphoric disorder in a randomly selected group of urban and rural women. Psychol. Med..

[2] Steiner M., Macdougall M., Brown E. (2003). The premenstrual symptoms screening tool (PSST) for clinicians. Arch. Womens Ment. Health.

[3] Hylan T.R., Sundell K., Judge R. (1999). The impact of premenstrual symptomatology on functioning and treatment-seeking behavior: Experience from the United States, United Kingdom, and France. J. Womens Health Gend. Based Med..

[4] Le J., Thomas N., Gurvich C. (2020). Cognition, the Menstrual Cycle, and Premenstrual Disorders: A Review. Brain Sci..

[5] Hara Y., Waters E.M., McEwen B.S., Morrison J.H. (2015). Estrogen Effects on Cognitive and Synaptic Health Over the Lifecourse. Physiol. Rev..

[6] Brinton R.D., Thompson R.F., Foy M.R., Baudry M., Wang J., Finch C.E., Morgan T.E., Pike C.J., Mack W.J., Stanczyk F.Z. (2008). Progesterone receptors: Form and function in brain. Front. Neuroendocr..

[7] Wallace M., Luine V., Arellanos A., Frankfurt M. (2006). Ovariectomized rats show decreased recognition memory and spine density in the hippocampus and prefrontal cortex. Brain Res..

[8] Engler-Chiurazzi E.B., Singh M., Simpkins J.W. (2016). Reprint of: From the 90’s to now: A brief historical perspective on more than two decades of estrogen neuroprotection. Brain Res..

[9] Woolley C.S., McEwen B.S. (1993). Roles of estradiol and progesterone in regulation of hippocampal dendritic spine density during the estrous cycle in the rat. J. Comp. Neurol..

[10] Sundström Poromaa I., Gingnell M. (2014). Menstrual cycle influence on cognitive function and emotion processing-from a reproductive perspective. Front. Neurosci..

[11] Voyer D., Voyer S., Bryden M.P. (1995). Magnitude of sex differences in spatial abilities: A meta-analysis and consideration of critical variables. Psychol. Bull..

[12] Dietrich T., Krings T., Neulen J., Willmes K., Erberich S., Thron A., Sturm W. (2001). Effects of blood estrogen level on cortical activation patterns during cognitive activation as measured by functional MRI. NeuroImage.

[13] Halari R., Hines M., Kumari V., Mehrotra R., Wheeler M., Ng V., Sharma T. (2005). Sex differences and individual differences in cognitive performance and their relationship to endogenous gonadal hormones and gonadotropins. Behav. Neurosci..

[14] Kozaki T., Yasukouchi A. (2009). Sex differences on components of mental rotation at different menstrual phases. Int. J. Neurosci..

[15] Bull J.R., Rowland S.P., Scherwitzl E.B., Scherwitzl R., Danielsson K.G., Harper J. (2019). Real-world menstrual cycle characteristics of more than 600,000 menstrual cycles. NPJ Digit. Med..

[16] Mumford S.L., Schisterman E.F., Gaskins A.J., Pollack A.Z., Perkins N.J., Whitcomb B.W., Ye A., Wactawski-Wende J. (2011). Realignment and multiple imputation of longitudinal data: An application to menstrual cycle data. Paediatr. Perinat. Epidemiol..

[17] Conklin S.E., Knezevic C.E. (2020). Advancements in the gold standard: Measuring steroid sex hormones by mass spectrometry. Clin. Biochem..

[18] Hamidovic A., Soumare F., Naveed A., Davis J., Sun J., Dang N. (2022). Reduced Dehydroepiandrosterone-Sulfate Levels in the Mid-Luteal Subphase of the Menstrual Cycle: Implications to Women’s Health Research. Metabolites.

[19] Endicott J., Nee J., Harrison W. (2005). Daily Record of Severity of Problems (DRSP): Reliability and validity. Arch. Womens Ment. Health.

[20] Hamidovic A., Davis J., Soumare F., Naveed A., Ghani Y., Semiz S., Khalil D., Wardle M. (2023). Allopregnanolone Is Associated with a Stress-Induced Reduction of Heart Rate Variability in Premenstrual Dysphoric Disorder. J. Clin. Med..

[21] Li H.J., Goff A., Rudzinskas S.A., Jung Y., Dubey N., Hoffman J., Hipolito D., Mazzu M., Rubinow D.R., Schmidt P.J. (2021). Altered estradiol-dependent cellular Ca2+ homeostasis and endoplasmic reticulum stress response in Premenstrual Dysphoric Disorder. Mol. Psychiatry.

[22] Hamidovic A., Soumare F., Naveed A., Davis J. (2023). Mid-Luteal Progesterone Is Inversely Associated with Premenstrual Food Cravings. Nutrients.

[23] Hampson E., Levy-Cooperman N., Korman J.M. (2014). Estradiol and mental rotation: Relation to dimensionality, difficulty, or angular disparity?. Horm. Behav..

[24] Vandenberg S.G., Kuse A.R. (1978). Mental rotations, a group test of three-dimensional spatial visualization. Percept. Mot. Skills.

[25] Hausmann M., Slabbekoorn D., Van Goozen S.H., Cohen-Kettenis P.T., Güntürkün O. (2000). Sex hormones affect spatial abilities during the menstrual cycle. Behav. Neurosci..

[26] Peters M., Laeng B., Latham K., Jackson M., Zaiyouna R., Richardson C. (1995). A redrawn Vandenberg and Kuse mental rotations test: Different versions and factors that affect performance. Brain Cogn..

[27] Jansen P., Heil M. (2009). Gender differences in mental rotation across adulthood. Exp. Aging Res..

[28] Courvoisier D.S., Renaud O., Geiser C., Paschke K., Gaudy K., Jordan K. (2013). Sex hormones and mental rotation: An intensive longitudinal investigation. Horm. Behav..

[29] Maki P.M., Rich J.B., Rosenbaum R.S. (2002). Implicit memory varies across the menstrual cycle: Estrogen effects in young women. Neuropsychologia.

[30] Leeners B., Kruger T.H.C., Geraedts K., Tronci E., Mancini T., Ille F., Egli M., Röblitz S., Saleh L., Spanaus K. (2017). Lack of Associations between Female Hormone Levels and Visuospatial Working Memory, Divided Attention and Cognitive Bias across Two Consecutive Menstrual Cycles. Front. Behav. Neurosci..

[31] Ghazal K., Brabant S., Prie D., Piketty M.-L. (2022). Hormone Immunoassay Interference: A 2021 Update. Ann. Lab. Med..

[32] Demers L.M. (2008). Testosterone and estradiol assays: Current and future trends. Steroids.

[33] Gauthier I., Hayward W.G., Tarr M.J., Anderson A.W., Skudlarski P., Gore J.C. (2002). BOLD activity during mental rotation and viewpoint-dependent object recognition. Neuron.

[34] Harris I.M., Egan G.F., Sonkkila C., Tochon-Danguy H.J., Paxinos G., Watson J.D. (2000). Selective right parietal lobe activation during mental rotation: A parametric PET study. Brain J. Neurol..

[35] Zacks J.M. (2008). Neuroimaging studies of mental rotation: A meta-analysis and review. J. Cogn. Neurosci..

[36] Epperson C.N., Haga K., Mason G.F., Sellers E., Gueorguieva R., Zhang W., Krystal J.H. (2002). Cortical gamma-aminobutyric acid levels across the menstrual cycle in healthy women and those with premenstrual dysphoric disorder: A proton magnetic resonance spectroscopy study. Arch. Gen. Psychiatry.

[37] Manyukhina V.O., Orekhova E.V., Prokofyev A.O., Obukhova T.S., Stroganova T.A. (2022). Altered visual cortex excitability in premenstrual dysphoric disorder: Evidence from magnetoencephalographic gamma oscillations and perceptual suppression. PLoS ONE.

[38] Christophel T.B., Cichy R.M., Hebart M.N., Haynes J.-D. (2015). Parietal and early visual cortices encode working memory content across mental transformations. NeuroImage.

[39] Salinas J., Mills E.D., Conrad A.L., Koscik T., Andreasen N.C., Nopoulos P. (2012). Sex differences in parietal lobe structure and development. Gend. Med..

[40] Koscik T., O’Leary D., Moser D.J., Andreasen N.C., Nopoulos P. (2009). Sex differences in parietal lobe morphology: Relationship to mental rotation performance. Brain Cogn..

[41] Studholme C., Kroenke C.D., Dighe M. (2020). Motion corrected MRI differentiates male and female human brain growth trajectories from mid-gestation. Nat. Commun..

[42] Dubol M., Stiernman L., Wikström J., Lanzenberger R., Epperson C.N., Sundström-Poromaa I., Bixo M., Comasco E. (2022). Differential grey matter structure in women with premenstrual dysphoric disorder: Evidence from brain morphometry and data-driven classification. Transl. Psychiatry.

